# Bilateral vestibulopathy in anti-IgLON5 disease

**DOI:** 10.1007/s00415-020-10386-5

**Published:** 2021-01-23

**Authors:** Christoph Helmchen, Klaus-Peter Wandinger, Armin Steffen, Thomas F. Münte, Norbert Brüggemann

**Affiliations:** 1 Department of Neurology, University Hospital Schleswig Holstein, University of Lübeck, Campus Lübeck, Ratzeburger Allee 160, 23538 Lubeck, Germany; 2 Institute of Clinical Chemistry, University Hospital Schleswig-Holstein, Lübeck, Germany; 3 Department of Otorhinolaryngology, University Hospital Schleswig-Holstein, Lübeck, Germany; 4 Institute of Neurogenetics, University of Lübeck, Lübeck, Germany; 5 Center of Brain, Behavior and Metabolism (CBBM), University of Lübeck, Ratzeburger Allee 160, 23562 Lübeck, Germany

Dear Sirs,

Anti-IgLON5 disease was first described in 2014 and is characterized by non-REM and REM parasomnias, obstructive sleep apnea and neurological manifestations affecting the central nervous system in association with antibodies against extracellular epitopes of IgLON5, a neuronal cell adhesion protein [3, 7]. IgLON5 antibodies are highly specific and the diagnosis can additionally be corroborated by the analysis of the human leukocyte antigens HLADRB1*10:01 and HLA-DQB1*05:01 which can be detected in 87% of patients [3, 6]. Neurological signs of the anti-IgLON5 disease [7] are manifold and comprise bulbar signs, a syndrome resembling progressive supranuclear palsy [1], cognitive decline, gait abnormalities and ataxia. Although signs of additional peripheral neuropathy have recently been reported in a few patients [8], vestibular dysfunction as a potential cause of gait unsteadiness has not been described previously in patients with IgLON5 antibodies.

Here, we report an IgLON5-antibody-positive 74-year-old woman with severe bilateral vestibulopathy (BV) suffering from long-standing (12 years) progressive gait unsteadiness. Gait instability increased with head movements and in darkness. She had noticed tingling and paresthesia in her lower legs for about the same time.

Follow-up clinical (head impulse test) and quantitative testing revealed severe progressive bilateral vestibulopathy and some clinical [reduced tendon reflexes in the legs, moderately reduced vibration sense at the ankles] but no neurographic evidence for polyneuropathy over years. Romberg test was pathological and she showed a moderately broad-based gait. Oculomotor and the rest of the neurological examination were normal.

Laboratory examinations were negative for routine work up for polyneuropathies, including cerebrospinal fluid. Strikingly, antibodies against IgLON5 (serum 1:10,000; CSF 1:32) were found as well as the IgLON5-associated HLADRB1*10:01/HLA-DQB1*05:01 haplotype. Additional antibody screening was negative for GABA_B_, GAD, LGI1, CASPR2, ANA, gangliosides GM1 and GM2, and GQ1b. Cancer screening was negative. Genetically, the *RFC1* pentanucleotide expansion was excluded ruling out CANVAS [5]. Screening for viral and bacterial agents remained negative except for IgG antibodies against the VlsE-antigen and IgM antibodies against the OspC antigen of Borrelia burgdorferi. In the absence of symptoms of a previous Lyme disease she was treated with doxycyclin but gait unsteadiness deteriorated.

**Fig. 1:**
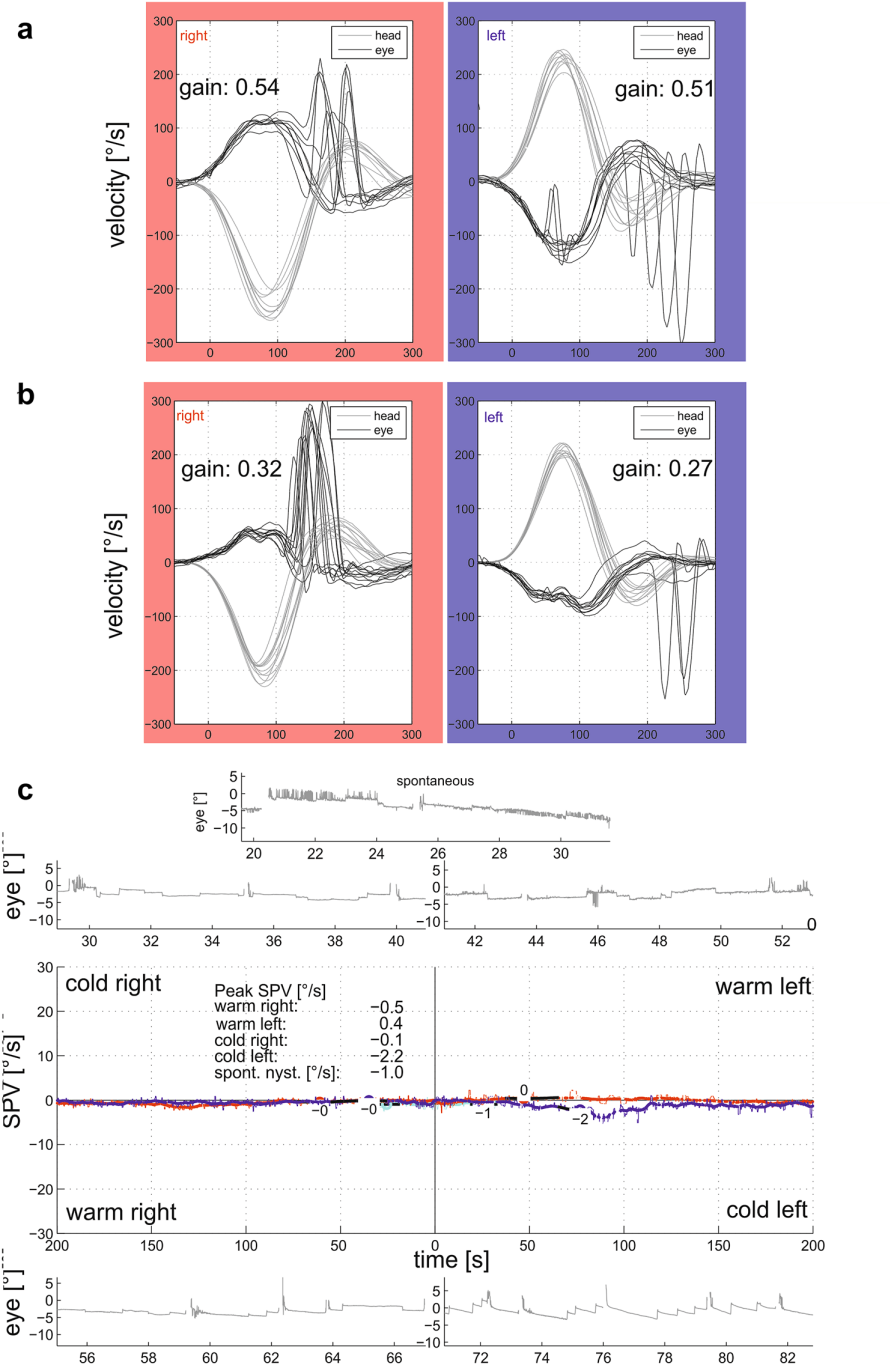
Quantitative head impulse testing (EyeSeeCam^®^) of the horizontal vestibulo-ocular reflex 6 (**a**) and 11 years (**b**) after symptom onset with progressive vestibular hypofunction and nearly absent responses to caloric irrigation (**c**), to chair rotation and absent ocular vestibular evoked myogenic potentials

Vestibular testing [10] revealed severe vestibular hypofunction with reduced gain of the vestibulo-ocular reflex (VOR) during quantitative head impulse testing, nearly absent responses to caloric irrigation (Fig. 1) and vestibular chair rotation and absent ocular vestibular evoked myogenic potentials. Subjective visual vertical was normal. Cranial MRI was normal. A type 4 home sleep test using peripheral arterial tone (WatchPAT^®^, Itamar) revealed an apnea/hypopnea index of 13.3/h (normal range: < 5/h) for the entire night, 26.6/h during REM sleep and 30.8/h for supine position [9]. The oxygen desaturation index was 6.2/h for the entire night (REM sleep: 15.8/h, supine position: 19.6/h, abnormal: > 15 events/h) with normal blood oxygen saturation (96%), and a reduced minimal oxygen saturation of 84% in this normal weighted patient (BMI 22 kg/m^2^). Sleep latency was normal (19 min), but shortened for REM sleep (29 min). The distribution of REM (23.9%) and deep sleep (22.2%) was normal. There were no signs of insomnia (sleep efficacy 88.3%, number of wakes per night 4). Daytime sleepiness was within normal limits (Epworth Sleepiness Scale 2 out of 24 points). Based on the detection of IgLON5 antibodies in 2019 she was initially treated with immunoglobulins and with rituximab for the last 3 months.

The combination of a high titer of antibodies against IgLON5 epitopes in serum and CSF, the HLA haplotype and sleep apnea and the exclusion of other known causes of BV makes it possible that BV in this patient reflects an additional sign of anti-IgLON5 disease. Post-mortem investigations of patients with anti-IgLON5 disease revealed the vestibular nuclei to contain the most extensive tau pathology in the brainstem [4], but peripheral vestibular nerve was not examined. Here, we provide some evidence that (i) BV is the major reason for gait unsteadiness in this patient with IgLON5 antibodies and that (ii) vestibular hypofunction is caused by peripheral vestibular organ damage. We, therefore, suggest that IgLON5 antibodies may damage the peripheral vestibular organ/nerve by a hitherto unknown mechanism which could reflect a previously unrecognized and potentially treatable reason for slowly progressive gait unsteadiness in “idiopathic” BV [2]. IgLON5-related BV may enlarge the multisystemic phenotype of this new disease.
